# Analyzing Key Predictors of Postoperative Delirium Following Coronary Artery Bypass Grafting and Aortic Valve Replacement: A Machine Learning Perspective

**DOI:** 10.3390/medicina61050883

**Published:** 2025-05-13

**Authors:** Marija Stošić, Velimir Perić, Dragan Milić, Milan Lazarević, Jelena Živadinović, Vladimir Stojiljković, Aleksandar Kamenov, Aleksandar Nikolić, Mlađan Golubović

**Affiliations:** 1 Clinic for Cardiac Surgery, University Clinical Center Nis, 18000 Nis, Serbia; velperic@gmail.com (V.P.); drdraganmilic@gmail.com (D.M.); serbvlada@yahoo.com (V.S.); kamenovcs@gmail.com (A.K.); mladjangolubovic@gmail.com (M.G.); 2 Faculty of Medicine, University of Nis, 18000 Nis, Serbia; dr_m.lazarevic@hotmail.com (M.L.); jelena5491@gmail.com (J.Ž.); draleksandarnikolic@hotmail.com (A.N.); 3 Institute Niska Banja, 18000 Nis, Serbia; 4 Clinic for Anesthesiology and Reanimatology, University Clinical Center Nis, 18000 Nis, Serbia

**Keywords:** postoperative delirium, coronary artery bypass, aortic valve replacement, predictive modeling, machine learning

## Abstract

*Background and Objectives*: Postoperative delirium (POD) is a frequent and severe complication following cardiac surgery, particularly in high-risk patients undergoing coronary artery bypass grafting (CABG) and aortic valve replacement (AVR). Despite extensive research, predicting POD remains challenging due to the multifactorial and often non-linear nature of its risk factors. This study aimed to improve POD prediction using an interpretable machine learning approach and to explore the combined effects of clinical, biochemical, and perioperative variables. *Materials and Methods*: This study included 131 patients who underwent CABG or AVR. POD occurrence was assessed using standard diagnostic criteria. Clinical, biochemical, and perioperative variables were collected, including patient age, sedation type, and mechanical ventilation status. Machine learning analysis was performed using an XGBoost classifier, with model interpretation achieved through SHapley Additive exPlanations (SHAP). Univariate logistic regression was applied to identify significant predictors, while SHAP analysis revealed variable interactions. *Results*: POD occurred in 34.3% of patients (n = 45). Patients who developed POD were significantly older (67.7 ± 6.5 vs. 64.5 ± 8.7 years, *p* = 0.020). Sedation with mechanical ventilation and the type of sedative used were strongly associated with POD (both *p* < 0.001). Sedation during mechanical ventilation showed the strongest association (OR = 2520.0; 95% CI: 80.9–78,506.7; *p* < 0.00001). XGBoost classifier achieved excellent performance (AUC = 0.998, accuracy = 97.6%, F1 score = 0.976). SHAP analysis identified sedation, mechanical ventilation, and their interactions with fibrinogen, troponin I, leukocyte parameters, and lung infection as key predictors. *Conclusions*: This study demonstrates that an interpretable machine learning approach can enhance POD prediction, providing insights into the combined impact of multiple clinical, biochemical, and perioperative factors. Integration of such models into perioperative workflows may enable early identification of high-risk patients and support individualized preventive strategies.

## 1. Introduction

Postoperative delirium (POD) is increasingly recognized as a critical outcome following major cardiac surgical procedures, particularly in high-risk populations undergoing complex interventions such as coronary artery bypass grafting (CABG) and aortic valve replacement (AVR). These procedures often necessitate extensive anesthesia and postoperative care, resulting in a notably high incidence of POD, with studies indicating rates ranging from 30% to 60% [1,2].

Clinically characterized by an acute and fluctuating disturbance in attention, awareness, and cognition, POD can emerge within hours to days following surgery, significantly complicating a patient’s recovery. The implications of POD are far-reaching, leading to increased morbidity and mortality, prolonged reliance on mechanical ventilation, lengthier stays in intensive care units (ICUs) and hospitals, and increased healthcare costs [3,4]. Moreover, patients who experience POD may suffer from long-term cognitive decline, highlighting the importance of recognizing and addressing this condition proactively [5].

Recent analyses have revealed a constellation of factors that contribute to the increased risk of POD in patients undergoing CABG and AVR. Among these factors, key predictors have been identified that warrant special attention due to their significant impact on patient outcomes. Notably, sedative management during the perioperative period plays a critical role in the development of POD. The type and duration of sedation can notably affect cognitive function, with deep levels of sedation being implicated in higher rates of delirium [6]. Additionally, prolonged mechanical ventilation, an everyday necessity in the immediate postoperative setting, can contribute to altered cognitive status and increase the risk of developing delirium [7]. Furthermore, specific strategies, such as glucose–insulin–potassium (GIK) infusion, have emerged as essential considerations in managing patients before and after cardiac surgery. GIK infusions can stabilize metabolic functions during the surgical procedure, potentially minimizing neuroinflammatory responses that may lead to POD. Research suggests that maintaining optimal glucose levels and preventing metabolic derangements can have a protective effect against delirium [8]. Fibrinogen levels have also been identified as a pertinent predictor of POD. Aberrant fibrinogen levels can reflect underlying inflammatory states, and elevated fibrinogen has been linked to postoperative complications, including delirium. Monitoring and managing fibrinogen levels could thus serve as a valuable component of delirium prevention strategies in high-risk cardiac patients [9]. Another significant predictor of POD is the incidence of lung infections during the postoperative period. Post-surgical lung infections can exacerbate systemic inflammation and lead to hypoxia, both of which are known to predispose patients to delirium [10]. The presence of pneumonia or other respiratory complications can quickly escalate into serious health issues, further complicating the clinical picture and leading to cognitive disturbances.

The pathophysiological mechanisms underlying postoperative delirium (POD) are complex and multifactorial, involving a combination of neuroinflammatory cascades, neurotransmitter imbalances, and pre-existing vulnerabilities. In this context, understanding these key predictors—sedation, mechanical ventilation, type of sedation, glucose—insulin–potassium infusion, fibrinogen levels, and lung infection—becomes essential for developing effective preventive measures and tailoring postoperative care for patients undergoing CABG and AVR.

Given the inherent challenges of POD prediction, conventional risk assessment methods often fall short, as they fail to account for the intricate, non-linear relationships among these predictors. This limitation underscores the urgent need for innovative approaches that can effectively synthesize and analyze vast amounts of clinical data. Machine learning (ML) methodologies, particularly advanced algorithms such as XGBoost, present a promising opportunity to enhance point-of-care (POC) predictions. These models are capable of processing large datasets and identifying complex interactions amongst variables that traditional models may overlook [11,12].

This study aims to utilize an optimized XGBoost model to enhance the prediction of postoperative delirium (POD) among patients undergoing coronary artery bypass grafting (CABG) and aortic valve replacement (AVR). By utilizing a comprehensive dataset that incorporates clinical, biochemical, and procedural variables, with a primary focus on the identified key predictors, we aim to develop a robust predictive tool that enhances the early identification of at-risk patients. Furthermore, to improve the clinical applicability and transparency of our model, we will employ SHapley Additive exPlanations (SHAP) to elucidate the contributions of individual features to the model’s predictions [13]. This approach aims to clarify how factors such as sedation types, mechanical ventilation practices, GIK infusion protocols, fibrinogen levels, and lung infection risks contribute to the likelihood of developing POD. By systematically identifying and ranking the most influential predictors of POD, this research intends to bridge the gap between data-driven modeling and real-world clinical practice. This enhanced predictive capability can empower healthcare providers to implement timely and tailored interventions, thereby reducing the incidence of POD and improving overall postoperative outcomes for patients undergoing CABG and AVR.

In conclusion, addressing the critical issue of postoperative delirium following CABG and AVR is vital for enhancing surgical outcomes and optimizing patient care. A deeper understanding of key predictors and their interplay will facilitate the implementation of targeted prevention strategies, ultimately enhancing health outcomes and improving the quality of life for cardiac surgery patients. Through rigorous validation of our machine learning models and their integration into clinical workflows, we aim to create a paradigm shift in how we approach the prevention and management of POD, thereby establishing a foundation for enhanced patient safety and quality of care.

## 2. Materials and Methods

### 2.1. Study Participants

This prospective, observational, single-center study included 131 patients who underwent elective or emergency cardiac surgery between May and September 2024 at the Clinic for Cardiac Surgery, University Clinical Center in Niš, Serbia. Surgical procedures included isolated coronary artery bypass grafting (CABG), aortic valve replacement (AVR) with mechanical prosthesis, and combined CABG + AVR procedures.

This study was conducted in accordance with the principles of the Declaration of Helsinki and was approved by the Ethics Committee of the Faculty of Medicine, University of Niš, and the Ethics Committee of the University Clinical Center Niš (approval number 38349/6, date: 15 December 2023). Informed written consent was obtained from all participants.

Patients were selected consecutively during routine clinical admission. Sample size was estimated based on an expected incidence of postoperative delirium (POD) of 35%, with a confidence level of 95%, statistical power of 80%, and an acceptable margin of error of 5%. The calculated minimum required sample size was 126 patients; the final enrolled cohort included 131.

The inclusion criteria were as follows: age ≥18 years, indication for isolated or combined CABG and/or AVR surgery, no evidence of preoperative cognitive dysfunction, and ability to provide informed consent. Exclusion criteria included preoperative cognitive impairment, history of stroke, significant carotid artery stenosis (>60%), or refusal to participate.

Preoperatively, all patients underwent comprehensive clinical assessment, including medical history, physical examination, laboratory testing, electrocardiography (ECG), and transthoracic echocardiography. Cognitive and psychological status were evaluated using the Mini-Mental State Examination (MMT) and the Hospital Anxiety and Depression Scale (HADS).

All procedures were performed under balanced general endotracheal anesthesia via median sternotomy. In patients requiring cardiopulmonary bypass (CPB), myocardial protection was achieved using cold crystalloid antegrade intermittent cardioplegia. Myocardial revascularization was performed with autologous internal thoracic artery and/or saphenous vein grafts, with or without CPB. Intraoperative cell salvage was routinely applied.

Postoperatively, patients were managed in the intensive care unit (ICU) under anesthesiologist supervision. Neurological complications, including POD, were monitored daily by trained ICU staff in collaboration with a neurologist. Neuroimaging (cranial CT angiography) was performed when indicated. POD was assessed daily using the Confusion Assessment Method for the Intensive Care Unit (CAM-ICU).

In addition to routine clinical monitoring, postoperative evaluation included serial analysis of laboratory biomarkers (including complete blood count, neutrophils, fibrinogen, troponin I, C-reactive protein, and D-dimer). Other variables recorded included the need for continuous administration of inotropes, vasopressors, insulin, or amiodarone, the amount of transfused blood and blood products, need for surgical reintervention, reintubation, use of non-invasive ventilation, prolonged mechanical ventilation, and sedation after extubation.

### 2.2. Statistical Analysis

Descriptive and comparative statistical analyses were performed using IBM SPSS Statistics, version 27 (IBM Corp., Armonk, NY, USA). Continuous variables were presented as mean ± standard deviation (SD), while categorical variables were reported as absolute frequencies and percentages. The normality of distribution was assessed using the Shapiro–Wilk test. Depending on data distribution, either the independent-samples Student’s *t*-test (for normally distributed variables) or the Mann–Whitney U test (for non-normally distributed variables) was used for comparison of continuous variables. The chi-square (χ^2^) test or Fisher’s exact test (for 2 × 2 contingency tables with small expected counts) was used to compare categorical variables. A two-tailed *p*-value less than 0.05 was considered statistically significant. Univariate logistic regression was performed to estimate the odds ratio (OR) and 95% confidence interval (CI) for the association between selected clinical variables and the occurrence of postoperative delirium (POD). For categorical predictors with more than two levels, each category was compared individually against the reference group. Multivariate logistic regression analysis was also performed to identify independent predictors of POD.

### 2.3. Machine Learning Methods

For machine learning analysis, all preprocessing, model training, and evaluation were performed in Python (version 3.10). Prior to model development, missing values were imputed using multivariate imputation by chained equations (MICE) for continuous variables and mode imputation for categorical variables. All continuous features were standardized using z-score normalization to ensure comparability across scales. To address class imbalance between POD and non-POD groups, the Synthetic Minority Oversampling Technique (SMOTE) was applied to the training set. The dataset was randomly split into training (80%) and testing (20%) subsets, ensuring stratification based on the target variable (POD occurrence) to maintain class proportions. Feature selection was guided by clinical relevance, univariate *p*-values, and intercorrelation analysis to reduce multicollinearity. An eXtreme Gradient Boosting (XGBoost) classifier was trained on the training set using the selected features. Model performance was evaluated on the unseen test set using accuracy, precision, recall, F1 score, and the area under the receiver operating characteristic curve (AUC). Additional internal validation was conducted using stratified 5-fold cross-validation within the training set to optimize model generalizability. To improve model transparency, SHapley Additive exPlanations (SHAP) analysis was employed. SHAP values were calculated to determine the global importance of each predictor and its local contribution to individual predictions. This approach enabled the identification of complex feature interactions and facilitated clinical interpretation of the model’s decision-making process. The primary objective of model development was to identify clinically meaningful predictors of POD rather than to optimize predictive performance alone, and overfitting was mitigated through stratified cross-validation and evaluation on an independent test set.

## 3. Results

Demographic, clinical, and other relevant patient characteristics for this study and the occurrence of POD are presented in Table S1 (Supplementary Material). Among the 131 patients included in this study, postoperative delirium (POD) was observed in 45 patients (34.3% of cases). Patients who developed POD were significantly older compared to those without POD (67.69 ± 6.54 vs. 64.50 ± 8.68 years, *p* = 0.0201). Inflammatory and immune-related markers also differed between groups: postoperative neutrophil count was significantly higher in the POD group (75.36 ± 11.33 vs. 69.64 ± 16.40, *p* = 0.021). Notably, sedation and mechanical ventilation were found to be highly associated with POD occurrence. All patients in the non-POD group had no history of sedation, whereas 4.4% of POD patients received mechanical ventilation and sedatives postoperatively (*p* < 0.001). Similarly, the use of sedatives was reflected in the “What sedative” score (0.00 ± 0.00 in non-POD vs. 2.50 ± 1.59 in the POD group, *p* < 0.001). This variable was originally categorical, indicating which sedative was used, and was recoded numerically for analysis purposes as follows: 0—no sedative; 1—haloperidol; 2—propofol; 3—midazolam; 4—diazepam (bensedin); and 5—dexmedetomidine. The score does not imply ordinal strength or duration, but was used to enable statistical comparison. The incidence of acute respiratory distress syndrome (ARDS) was also significantly higher among POD patients (13.3% vs. 0%, *p* = 0.0025).

Several variables showed no statistical significance but are clinically noteworthy. Fibrinogen levels were slightly elevated in the POD group (4.59 ± 0.97 vs. 4.42 ± 0.80, *p* = 0.3114), suggesting a possible proinflammatory tendency. The use of vasopressors was more prolonged among POD patients (33.44 ± 44.78 vs. 20.90 ± 24.58 min), although this difference did not reach statistical significance (*p* = 0.0859). ASPI test results indicated higher platelet aggregation in POD patients (743.96 ± 349.99 vs. 629.24 ± 352.44, *p* = 0.0789), potentially reflecting altered platelet reactivity. There were no significant differences between groups in terms of gender distribution, body mass index, baseline systolic and diastolic blood pressures, or comorbidities, such as diabetes, hypertension, or chronic obstructive pulmonary disease (COPD). In univariate analysis, sedation during mechanical ventilation (Sedation MV) showed the strongest association with POD (OR = 2520.0; 95% CI: 80.9–78,506.7; *p* < 0.00001). Additionally, the type of administered sedative (What sedative) was significantly associated with POD occurrence. Compared to patients who did not receive sedation (category 0), the use of haloperidol, propofol, and dexmedetomidine (categories 1, 2, and 5) was associated with markedly increased odds of delirium (ORs ranging from 672.0 to 2520.0; all *p* < 0.00001). We initially attempted multivariate logistic regression to identify independent predictors of postoperative delirium (POD). However, the model proved to be unstable due to significant multicollinearity among several variables, as well as perfect separation in key predictors such as sedation with mechanical ventilation. These issues led to convergence problems and unreliable estimates, rendering the multivariate approach inappropriate for this dataset. Given these limitations, we proceeded with a machine learning approach (XGBoost) capable of handling complex feature interactions and multicollinearity, while providing interpretable insights through SHAP analysis.

**Figure 1:**
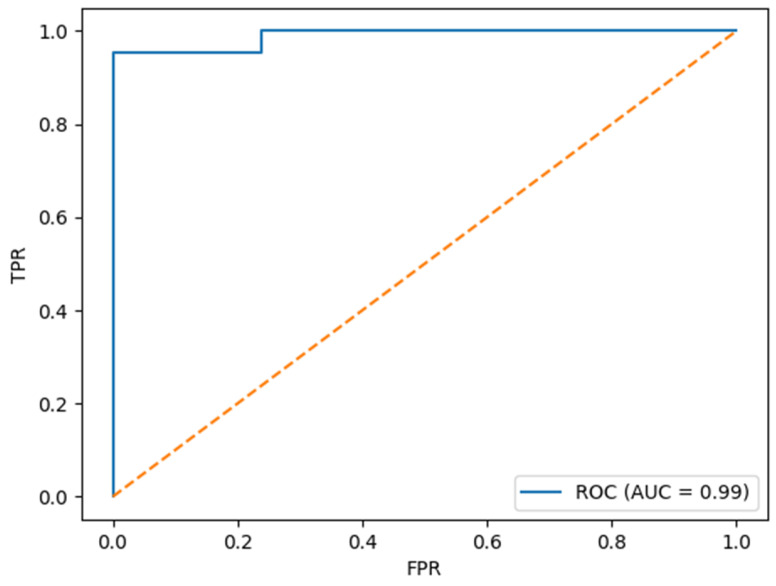
ROC curve for obtained XGBoost model.

In addition to conventional statistical analysis, we developed a predictive model using the XGBoost algorithm to classify patients at risk for postoperative delirium. The model demonstrated excellent performance in internal validation, with an accuracy of 97.6%, an area under the receiver operating characteristic curve (AUC) of 0.998, a precision of 1.00, a recall (sensitivity) of 0.95, and an F1 score of 0.976. The obtained ROC curve is presented in Figure 1.

**Figure 2:**
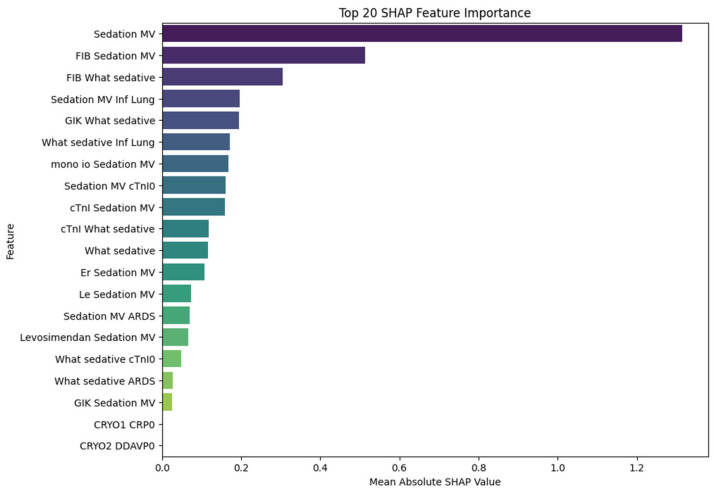
Top 20 features ranked by mean absolute SHAP value, indicating their overall importance in the XGBoost model for predicting postoperative delirium. The x-axis represents the mean absolute SHAP value, quantifying each feature’s average contribution to the model output. Features on the y-axis are sorted from most to least important. Higher SHAP values correspond to a stronger influence on the prediction, regardless of direction (positive or negative). This plot summarizes global feature importance across all patients in the cohort.

These results suggest that the model not only achieves high discriminative ability but also maintains a favorable balance between sensitivity and precision, making it a potentially valuable tool for the early identification of high-risk patients. Notably, the high AUC value underscores the model’s robustness in distinguishing between POD and non-POD cases across varying thresholds. In line with these findings, variables related to sedation, inflammatory status, and cardiac biomarkers emerged as the most important predictors in the subsequent XGBoost model, as shown in the SHAP analysis and presented in Figure 2. Notably, features such as sedation with mechanical ventilation, fibrinogen levels, troponin I, and the presence of lung infection showed the highest SHAP values, suggesting a substantial contribution to delirium risk classification.

**Figure 3:**
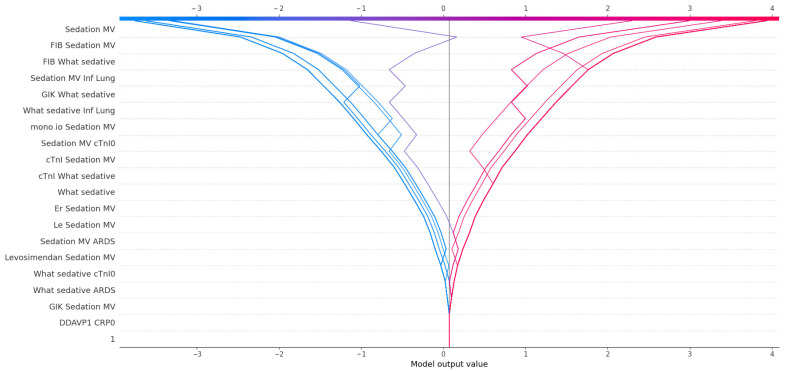
The SHAP decision plot shows individual features’ cumulative impact on the XGBoost model’s output for postoperative delirium prediction. Each line represents a single patient, with the x-axis indicating the model output value (log-odds scale), and the y-axis listing the top 20 contributing features. Lines diverge from zero based on the additive SHAP values of the most influential features. Color represents directionality, with red indicating positive contributions toward predicted delirium and blue indicating negative contributions. This visualization highlights how combinations of interacting predictors shape individual risk profiles.

To further explore the predictors of postoperative delirium (POD), we applied SHAP (SHapley Additive exPlanations) analysis to the trained XGBoost model and impact of SHAP values on model outcome is presented in Figure 3. The feature with the highest mean absolute SHAP value was sedation with mechanical ventilation, confirming its central role in POD prediction and reinforcing the strong statistical association observed in classical analyses. Beyond its standalone impact, sedation also appeared in numerous high-impact interactions. For instance, fibrinogen (FIB), although not independently statistically significant (*p* = 0.31), showed substantial importance when combined with sedation variables, suggesting a synergistic effect between coagulation activity and sedation-related systemic stress. Similarly, What sedative, a variable quantifying exposure to sedatives, was both an independent contributor and a frequent partner in interaction terms—most notably with lung infection (Inf Lung), cTnI, and GIK infusion. These combinations may reflect additive effects of pharmacologic, inflammatory, and metabolic stress on neural vulnerability.

Cardiac biomarkers also emerged as relevant predictors. Troponin I (cTnI) and cTnI0 were not significantly different between groups in univariate analysis, yet their interactions with sedation and GIK carried predictive weight. This implies that myocardial stress, although not sufficient alone, may contribute to POD risk when it coincides with systemic or pharmacological disturbances. Markers of immune activation, such as mono io and leukocyte count (Le), gained importance primarily through their interaction with sedation. The model assigned meaningful SHAP values to combinations such as mono-IO + Sedation MV and Le + Sedation MV, indicating a link between subclinical immune activation and susceptibility to neurologic dysfunction under critical care stress.

Interestingly, levosimendan appeared in SHAP interactions despite lacking statistical significance, suggesting that inotropic support may subtly modulate POD risk, possibly through hemodynamic effects or its interaction with the depth of sedation. The variable ARDS, although rare, also contributed to the model through its co-occurrence with sedation and pharmacologic variables. In clinical terms, this could reflect a more severe disease trajectory marked by hypoxia, inflammation, and increased sedative load.

Taken together, SHAP analysis provided a nuanced view of POD risk, emphasizing not only the dominant influence of sedation and mechanical ventilation but also the latent contribution of biomarkers, immune status, and pharmacologic interactions. These findings underscore the multidimensional pathophysiology of delirium and the value of interpretable machine learning in capturing such complexity.

## 4. Discussion

Postoperative delirium (POD) remains a prevalent and clinically significant complication following cardiac surgery, with far-reaching consequences for patient recovery, length of stay, and long-term cognitive outcomes. In our cohort of patients undergoing coronary artery bypass grafting (CABG) and aortic valve replacement (AVR), POD was observed in 34.3% of cases, which aligns with the existing literature for this high-risk population [14,15].

Several clinical features traditionally associated with POD were confirmed in our analysis. Age was significantly higher in patients who developed delirium, reinforcing its role as a well-established risk factor linked to reduced cognitive reserve and increased neuroinflammatory sensitivity [16]. Elevated neutrophil counts in the POD group suggest that systemic inflammation may play a role in delirium pathogenesis, potentially through cytokine-mediated neurotoxicity or indirectly via tissue injury and metabolic disruption [17]. Most notably, sedation and mechanical ventilation were strongly associated with the development of POD. Patients in the POD group were more frequently exposed to sedatives, while none of the non-POD patients required mechanical ventilation or sedative administration. This observation corroborates current pathophysiological models, indicating that sedation, particularly within the context of mechanical ventilation, contributes to altered neurotransmission, impaired oxygenation, and increased vulnerability of brain circuits associated with attention, arousal, and memory [18,19].

Other factors, although not statistically significant, remain clinically relevant. Fibrinogen levels and ASPI test values were higher in POD patients, potentially reflecting an underlying prothrombotic or proinflammatory state that, while not sufficient to predict delirium independently, may contribute to a cumulative risk profile [20]. Similarly, prolonged vasopressor use, though not statistically significant, may indicate intraoperative hemodynamic instability or postoperative low-output syndrome—both of which can exacerbate cerebral perfusion vulnerability [21].

To better understand these nuanced relationships, we employed an XGBoost machine learning model with SHAP (SHapley Additive exPlanations) analysis for interpretability. Machine learning (ML) algorithms are increasingly being applied in perioperative risk prediction due to their ability to model complex, non-linear relationships among clinical variables. In contrast to traditional regression methods, which assume linearity and independence among predictors, gradient-boosting algorithms such as XGBoost iteratively optimize decision trees to capture subtle patterns and interactions that might otherwise remain undetected. This makes XGBoost particularly suitable for high-dimensional, heterogeneous clinical data commonly encountered in real-world patient populations. However, one of the primary limitations of complex ML models has traditionally been their interpretability. To address this, explainable AI techniques such as SHAP (SHapley Additive exPlanations) have been developed to consistently and intuitively quantify the contribution of each feature to the model’s output. SHAP values offer both local interpretability, explaining individual predictions, and global interpretability by ranking feature importance across the dataset. By applying SHAP analysis, researchers and clinicians can move beyond the binary output of a predictive model and instead gain insights into why a given patient is at elevated risk. This not only enhances clinical trust in the model’s outputs but also facilitates the generation of hypotheses and the identification of potential intervention targets. Importantly, SHAP can also uncover interaction effects and conditional dependencies that may not be evident in traditional univariate or multivariate analysis. Together, the use of XGBoost and SHAP represents a powerful paradigm for clinical prediction, combining high predictive performance with interpretable outputs that bridge the gap between data science and bedside decision-making [22].

SHAP results confirmed many key findings observed in classical statistics, particularly the dominant role of sedation with mechanical ventilation as the most influential predictor of postoperative day (POD). However, SHAP analysis further revealed a series of interactions and non-linear effects, providing a richer and more clinically intuitive framework for understanding risk. For instance, fibrinogen levels—statistically nonsignificant on their own—showed a substantial impact on POD prediction when combined with sedation-related features. This suggests that fibrinogen may function as a conditional risk factor, amplifying vulnerability only in the presence of systemic stressors such as sedation or inflammation [23]. Similarly, the variable “What sedative” emerged as a strong standalone predictor and frequently interacted with other factors, including lung infection, troponin I, and glucose–insulin–potassium (GIK) infusion. These multidimensional associations highlight the complex interplay between pharmacologic, metabolic, and inflammatory stressors in precipitating POD [24].

Cardiac biomarkers, such as troponin I and its early postoperative counterpart (cTnI0), also contributed to the prediction of POD risk within the machine learning framework despite their lack of significance in univariate analysis. Their importance became evident in interactions with sedation and other markers of physiological stress, implying that myocardial injury may act as a contextual vulnerability factor for POD [25]. Interestingly, markers of immune activation, such as leukocyte count and monocyte count, were identified by SHAP analysis as meaningful contributors to POD risk. This supports emerging theories suggesting that subclinical immune modulation may lower the threshold for developing delirium in critically ill patients [26]. Notably, the use of levosimendan, while not significant in traditional analysis, featured in influential SHAP interactions, indicating that hemodynamic modulation or catecholamine-sparing effects may influence neurologic resilience [27]. The presence of acute respiratory distress syndrome (ARDS), although infrequent, contributed to the model when interacting with sedation and pharmacologic factors, potentially reflecting compounded effects of hypoxia, systemic inflammation, and sedative load [28].

In our cohort, none of the patients underwent concomitant carotid artery stenting (CAS). However, patients undergoing coronary artery bypass grafting (CABG) often present with complex atherosclerotic disease, which may include significant carotid artery involvement. Although CAS was not a factor in our study, it remains a clinically relevant consideration, particularly given the potential impact of carotid pathology on cerebral perfusion and neurological outcomes. While our exclusion criteria accounted for high-grade carotid stenosis, we acknowledge that future research should further explore how coexisting vascular comorbidities, such as carotid disease and its interventional management, may influence the incidence and severity of postoperative delirium.

Overall, SHAP-based interpretation facilitated a nuanced and clinically realistic understanding of POD risk by highlighting non-linear relationships and synergistic patterns between variables. Compared to conventional methods that emphasize isolated predictors, interpretable machine learning promotes a shift toward multifactorial, context-aware risk stratification, which is particularly valuable in the intricate perioperative environment of cardiac surgery [22].

## 5. Conclusions

In conclusion, this study underscores the complex and multifactorial nature of postoperative delirium (POD) in patients undergoing coronary artery bypass grafting (CABG) and aortic valve replacement (AVR) [29,30]. Our findings identify sedation and mechanical ventilation as significant contributors to POD, corroborating previous research that links age and inflammatory markers to increased risk. Notably, the use of interpretable machine learning techniques has shed light on the intricate interactions among variables, such as fibrinogen, troponin, leukocyte counts, and pharmacological exposures, revealing additional layers of complexity that were not previously explored.

The implementation of SHAP analysis has proven invaluable, illustrating how variables that may individually exhibit limited predictive power can gain greater significance when combined. This approach advocates for a more nuanced, context-sensitive strategy in POD risk stratification, seamlessly integrating machine learning tools into existing perioperative risk assessment frameworks. Importantly, these tools should be viewed as complementary to clinical judgment, enhancing decision-making by uncovering hidden patterns that can inform personalized interventions.

However, several limitations warrant consideration. This study was conducted in a single tertiary center and included a modest sample size, which may restrict the generalizability of our findings to broader populations. Although machine learning models such as XGBoost are well suited to capturing non-linear, high-order interactions among clinical features, their performance is sensitive to data sparsity, potential overfitting, and class imbalance—factors that warrant caution in interpreting model strength outside this context. Furthermore, while SHAP analysis enhances transparency by attributing importance to individual features, it provides post hoc explanations that reflect statistical associations rather than causal mechanisms. As such, the observed variable contributions and interactions should not be interpreted as deterministic or pathophysiological evidence without further confirmatory studies.

Another important limitation lies in the clinical assessment of delirium. Despite the use of standardized diagnostic tools (CAM-ICU), detection and documentation may vary depending on the vigilance and training of clinical staff, particularly in patients with hypoactive or fluctuating symptoms. Additionally, our analysis did not incorporate certain potentially relevant intraoperative and baseline variables, including depth of anesthesia, EEG monitoring, or preoperative cognitive testing beyond MMT.

Finally, external validation of the model was not performed. Although our findings demonstrate strong internal predictive accuracy (AUC = 0.998), independent validation on multicenter cohorts is essential before this model can be generalized or integrated into clinical workflows.

These limitations outline clear avenues for future research. Large-scale, prospective, multicenter studies should aim to replicate and refine the observed relationships, particularly the interplay between sedation practices, systemic inflammation, and cardiac injury markers such as troponin I. Additionally, expanding variable sets to include intraoperative neurophysiological monitoring and long-term cognitive outcomes could enhance both prediction and mechanistic understanding. Ultimately, integrating interpretable machine learning tools into real-time perioperative risk assessment may support early identification of vulnerable patients and facilitate timely, individualized intervention strategies to reduce the burden of postoperative delirium.

## Data Availability

The data that support the findings of this study are available on request from the corresponding author.

## Supplementary Materials

The following supporting information can be downloaded at https://www.mdpi.com/article/10.3390/medicina61050883/s1: Table S1. Demographic, clinical, and other relevant patient characteristics for this study and the occurrence of POD.

## References

[1] Krohn C., Jaarsma T., Abu-Saad H.H., Dracup K. (2017). The prevalence and risk factors of delirium in cardiac surgery patients: A systematic review. Eur. J. Cardiovasc. Nurs..

[2] Witlox J., Eurelings L.S.M., de Jonghe J.F.M., Kalisvaart K.J., Eikelenboom P., van Gool W.A. (2010). Delirium in elderly patients: A systematic review. Lancet.

[3] Saczynski J.S., Marcantonio E.R., Quach L., Fong T.G., Gross A., Inouye S.K., Jones R.N. (2012). The effect of delirium on long-term cognitive decline. Arch. Intern. Med..

[4] Ely E.W., Inouye S.K., Bernard G.R., Gordon S., Francis J., May L., Truman B., Speroff T., Gautam S., Margolin R. (2001). Delirium in mechanically ventilated patients: Validity and reliability of the confusion assessment method for the intensive care unit (CAM-ICU). JAMA.

[5] Dyer C.B., Ashton C.M., Teasdale T.A. (2014). Postoperative delirium: A review of 80 primary data-collection studies. Alzheimers Dement..

[6] Krewulak K.D., Stelfox H.T., Leigh J.P., Ely E.W., Fiest K.M. (2019). Postoperative delirium in cardiac surgery: A review of current evidence and future directions. J. Card. Surg..

[7] Huang M., Lv Y., Huang K., Wang S., Wang W., Luo A. (2017). Impact of mechanical ventilation on the development of delirium in cardiac surgery patients: A systematic review. Crit. Care Med..

[8] Tsai S.L., Sands L.P., Leung J.M. (2016). Glucose-insulin-potassium infusion and its effects on delirium after cardiac surgery: A systematic review. Crit. Care Med..

[9] Hassan G.S., Abou-Hashem R.M., El-Masry A.G., Elhady A.M. (2019). Fibrinogen as a predictor of postoperative delirium in cardiac surgery: A cohort study. J. Thorac. Cardiovasc. Surg..

[10] Martin D.S., Grocott M.P.W. (2020). Postoperative lung infection and its impact on delirium in older patients: A review. Ann. Thorac. Surg..

[11] Boehm L.M., Dietrich M.S., Hensley S.J., Boehm M.W., Ely E.W., Pandharipande P.P., Girard T.D. (2020). Machine learning and delirium: A review of the literature. Curr. Neurosci. Res..

[12] Pakhare A., Makhija N., Gupta S., Garg R., Choudhury M., Das S., Choudhury A., Bhargava A. (2021). Predictive modeling of postoperative delirium: A comparison of conventional and machine learning approaches. BMC Anesthesiol..

[13] Lundberg S.M., Lee S.I. A unified approach to interpreting model predictions. Proceedings of the 31st International Conference on Neural Information Processing Systems.

[14] Marx J.A., Hockberger R.S., Walls R.M. (2019). Postoperative outcomes in patients undergoing cardiac surgery: A review. J. Am. Coll. Cardiol..

[15] Radtke F.M., Franck M., Lindner J., Krüger S., Wernecke K.D., Spies C. (2013). Incidence and risk factors of delirium following coronary artery bypass grafting. Anesth. Analg..

[16] Shenkin S.D., Russ T.C., Ryan T.M., MacLullich A.M.J. (2019). Cognitive reserve and its role in delirium. BMC Geriatr..

[17] Caplan G.A., Coconis J., Board N., Sayers A., Woods J. (2021). Inflammation and postoperative delirium: A practical approach. Anesth. Analg..

[18] Gropper M.A., Miller R.D., Eriksson L.I., Fleisher L.A., Wiener-Kronish J.P., Cohen N.H., Leslie K. (2017). The impact of sedation on delirium during cardiac surgery. Anesthesiology.

[19] Hsieh T.T., Inouye S.K., Oh E.S. (2015). The Role of Sedation in Developing Delirium. Arch. Intern. Med..

[20] Zhu J., Wang X., Shi H., Su W., Chen X., Wang Y., Zhang W., Wang Y. (2021). Inflammatory Markers and the Risk of Delirium in Cardiac Surgery: A Systematic Review. Cardiol. Clin..

[21] Yang L., Sun D.F., Han J., Liu R., Wang L.J., Zhang Z.Z. (2016). Effects of intraoperative hemodynamics on the incidence of postoperative delirium in elderly patients: A retrospective study. Med. Sci. Monit..

[22] Mateussi N., Rogers M.P., Grimsley E.A., Read M., Parikh R., Pietrobon R., Kuo P.C. (2024). Clinical applications of machine learning. Ann. Surg. Open.

[23] Jiang L., Lei G. (2022). Albumin/fibrinogen ratio, an independent risk factor for postoperative delirium after total joint arthroplasty. Geriatr. Gerontol. Int..

[24] Whitlock E.L., Vannucci A., Avidan M.S. (2011). Postoperative delirium. Minerva Anestesiol..

[25] Oshima K., Kunimoto F., Takahashi T., Mohara J., Takeyoshi I., Hinohara H., Okawa M., Saito S. (2010). Postoperative cardiac troponin I (cTnI) level and its prognostic value for patients undergoing mitral valve surgery. Int. Heart J..

[26] Ritter C., Tomasi C.D., Dal-Pizzol F., Pinto B.B., Dyson A., de Miranda A.S., Comim C.M., Soares M., Teixeira A.L., Quevedo J. (2014). Inflammation biomarkers and delirium in critically ill patients. Crit. Care.

[27] Harrison R.W., Hasselblad V., Mehta R.H., Levin R., Harrington R.A., Alexander J.H. (2013). Effect of levosimendan on survival and adverse events after cardiac surgery: A meta-analysis. J. Cardiothorac. Vasc. Anesth..

[28] Sasannejad C., Ely E.W., Lahiri S. (2019). Long-term cognitive impairment after acute respiratory distress syndrome: A review of clinical impact and pathophysiological mechanisms. Crit. Care.

[29] Othman S.M.A., Aziz M.A.A., Sriwayyapram C., Xu Q. (2024). Systematic literature review on early detection of postoperative delirium in adult patients after cardiac surgery. J. Cardiothorac. Surg..

[30] Wang Y., Wang B. (2024). Risk factors of delirium after cardiac surgery: A systematic review and meta-analysis. J. Cardiothorac. Surg..

