# Supplementary material for: Analyzing Key Predictors of Postoperative Delirium Following Coronary Artery Bypass Grafting and Aortic Valve Replacement: A Machine Learning Perspective
Source: Medicina (Kaunas). 2025 May 13;61(5):883. doi: 10.3390/medicina61050883 (PMC12113078; doi:10.3390/medicina61050883)
Supplement: Supplementary file 1 [file medicina-61-00883-s001.zip › medicina-3598707-supplementary.pdf]

Table S1. Demographic, clinical, and other relevant patient characteristics for this study and the occurrence of POD

Variable	Without POD	With POD	p-value
Gender	Male: 77.9%; Female: 22.1%	Male: 71.1%; Female: 28.9%	0.5185
Age	64.50± 8.68	67.69± 6.54	0.0201
Weight	81.16± 13.02	79.50± 15.23	0.5375
Height	168.50± 8.30	166.87± 9.61	0.3366
BSA	1.94± 0.19	1.91± 0.21	0.5273
BMI	28.63± 4.52	28.51± 4.69	0.8887
ASPI	629.24± 352.44	743.96± 349.99	0.0789
ADP	655.42± 263.77	652.38± 276.56	0.9517
TRAP	1218.28± 275.60	1210.56± 232.92	0.8661
INR	1.18± 1.01	1.07± 0.08	0.2979
aPTT	31.78± 6.34	30.81± 4.19	0.2986
PT	542.10± 9.31	11.74± 0.89	0.3201
FIB	4.42± 0.80	4.59± 0.97	0.3114
AT III	90.74± 11.69	90.69± 12.39	0.9803
Hgb	137.15± 13.43	136.38± 15.05	0.7728
Er	6.26± 15.15	4.61± 0.54	0.3179
Le	7.29± 2.25	7.59± 1.97	0.4389
Neutro	70.28± 67.88	63.40± 8.49	0.3571
Mono	6.08± 6.74	5.56± 2.15	0.5091
Tr	208.58± 46.49	221.13± 57.80	0.2119
Hct	0.87± 4.31	0.40± 0.04	0.32
PreSys	127.81± 17.00	131.13± 17.56	0.3018
PreDy	75.42± 11.09	72.09± 19.23	0.2881
PreSF	67.34± 9.14	66.20± 11.39	0.5641
Urea	6.89± 6.79	6.83± 2.15	0.9437
Creat	94.50± 22.24	93.84± 19.82	0.8613
ClCr Cocroft Gault	80.36± 23.51	73.24± 16.60	0.0526
Gluc	6.79± 2.42	6.61± 1.79	0.6214
Holes	4.14± 1.27	4.16± 1.07	0.9229
TGC	1.71± 1.12	1.60± 1.01	0.566
HDL	1.51± 0.89	4.57± 20.96	0.3316
LDL	527.90± 4.50	2.07± 1.05	0.3203
Bily	12.58± 8.46	11.84± 5.88	0.5621
Album	43.42± 3.31	43.06± 3.10	0.5345
CK MB	18.31± 10.77	16.78± 5.77	0.2898
cTnI	0.11± 0.72	0.17± 0.92	0.7282
cTnI0	4.46± 5.85	4.57± 4.70	0.9112
BNP	298.73± 530.42	332.18± 447.00	0.7041
CRP	4.75± 8.62	4.77± 5.39	0.988

Uric	314.47± 78.33	312.02± 80.41	0.8717
SAP	No:78.8%; Yes:21.2%	No:82.2%; Yes:17.8%	0.8177
NSAP	Yes:52.3%; No:47.7%	Yes:53.3%; No:46.7%	1
LMS	No:69.8%; Yes:30.2%	No:84.4%; Yes:15.6%	0.104
IM	Yes:70.9%; No:29.1%	Yes:64.4%; No:35.6%	0.5742
Prior PCI	No:76.7%; Yes:23.3%	No:88.9%; Yes:11.1%	0.1483
StenN	0.33± 0.85	0.18± 0.61	0.2551
TimeCE	16.67± 55.75	19.44± 44.82	0.7585
HTA	Yes:91.9%; No:8.1%	Yes:95.6%; No:4.4%	0.667
DM	No:69.8%; Yes:30.2%	No:71.1%; Yes:28.9%	1
DMID	No:87.2%; Yes:12.8%	No:91.1%; Yes:8.9%	0.7061
HypLip	Yes:59.3%; No:40.7%	Yes:55.6%; No:44.4%	0.821
Smoking	No:62.8%; Yes:37.2%	No:51.1%; Yes:48.9%	0.2701
Family history cardiac	No:30.2%; Yes:69.8%	No:26.7%; Yes:73.3%	0.8874
HBI	No:91.9%; Yes:8.1%	No:95.6%; Yes:4.4%	0.667
Cancer	No:95.3%; Yes:4.7%	No:97.8%; Yes:2.2%	0.8345
Inf Lung	No:97.6%; Yes:2.4%	No:93.3%; Yes:6.7%	0.4609
Hypothyreosis	No:94.2%; Yes:5.8%	No:93.3%; Yes:6.7%	1
HOBP	No:82.6%; Yes:17.4%	No:77.8%; Yes:22.2%	0.6693
PVB	No:88.4%; Yes:11.6%	No:91.1%; Yes:8.9%	0.8539
Levosimendan	No:86.0%; Yes:14.0%	No:91.1%; Yes:8.9%	0.5757
StenCar	No:88.4%; Yes:11.6%	No:86.7%; Yes:13.3%	0.9983
Sedative	No:91.9%; Yes:8.1%	No:88.9%; Yes:11.1%	0.8096
Neutro io	69.64± 16.40	75.36± 11.33	0.021
mono io	3.35± 1.30	1012.17± 6766.11	0.3227
Neu mon io	32.61± 45.56	33.18± 61.29	0.9569
IO INO	Yes:57.0%; No:43.0%	Yes:55.6%; No:44.4%	1
IO VP	Yes:76.7%; No:23.3%	Yes:75.6%; No:24.4%	1
IO levosimendan	No:84.9%; Yes:15.1%	No:86.7%; Yes:13.3%	0.9889
IO milrinon	No:97.7%; Yes:2.3%	No:100.0%	0.779
IO kortiko	Yes:70.6%; No:29.4%	Yes:66.7%; No:33.3%	0.794
IO amiodaron	No:61.6%; Yes:38.4%	No:53.3%; Yes:46.7%	0.466
GIK	No:93.0%; Yes:7.0%	No:91.1%; Yes:8.9%	0.9641
Time INO	24.13± 32.36	22.84± 23.67	0.7964
Time VP	20.90± 24.58	33.44± 44.78	0.0859
Time Ins pump	3.24± 11.79	6.96± 26.40	0.3735
Sedation MV	No:100.0%	Yes:95.6%; No:4.4%	0
What sedative	0.00± 0.00	2.50± 1.59	0
ARDS	No:100.0%	No:86.7%; Yes:13.3%	0.0025

Gender – Biological sex (male/female); **Age** – Patient age in years; **Weight** – Body weight (kg); **Height** – Body height (cm); **BSA** – Body surface area calculated using the Mosteller formula (m^2); **BMI** – Body mass index (kg/m^2); **ASPI** – Platelet aggregation induced by arachidonic acid (aggregation units); **ADP** – Platelet aggregation induced by adenosine diphosphate (aggregation units); **TRAP** – Platelet aggregation

induced by thrombin receptor-activating peptide (aggregation units); **INR** – International normalized ratio; **aPTT** – Activated partial thromboplastin time (seconds); **PT** – Prothrombin time (seconds); **FIB** – Fibrinogen concentration (g/L); **AT III** – Antithrombin III activity (%); **Hgb** – Hemoglobin (g/dL); **Er** – Erythrocyte (red blood cell) count ($\times10^{12}$/L); **Le** – Leukocyte (white blood cell) count ($\times10^{9}$/L); **Neutro** – Neutrophil count ($\times10^{9}$/L); **Mono** – Monocyte count ($\times10^{9}$/L); **Tr** – Platelet count ($\times10^{9}$/L); **Hct** – Hematocrit (%). **PreSys** – Preoperative systolic blood pressure (mmHg); **PreDy** – Preoperative diastolic blood pressure (mmHg); **PreSF** – Preoperative ejection fraction (%); **Urea** – Blood urea nitrogen (mmol/L); **Creat** – Serum creatinine (µmol/L); **ClCr Cocroft Gault** – Creatinine clearance estimated using the Cockcroft-Gault formula (mL/min); **Gluc** – Serum glucose (mmol/L); **Holes** – Serum sodium (Na^{+}) concentration (mmol/L); **TGC** – Triglycerides (mmol/L); **HDL** – High-density lipoprotein cholesterol (mmol/L); **LDL** – Low-density lipoprotein cholesterol (mmol/L); **Bily** – Total bilirubin (µmol/L); **Album** – Serum albumin (g/L); **CK MB** – Creatine kinase MB isoenzyme (U/L); **cTnI** – Postoperative cardiac troponin I (ng/mL); **cTnI0** – Preoperative cardiac troponin I (ng/mL); **BNP** – B-type natriuretic peptide (pg/mL); **CRP** – C-reactive protein (mg/L); **Uric** – Serum uric acid (µmol/L). **SAP** – Stable angina pectoris; **NSAP** – Non-stable angina pectoris; **LMS** – Left main coronary artery stenosis; **IM** – Inferior myocardial infarction; **Prior PCI** – History of percutaneous coronary intervention; **StenN** – Number of significant coronary stenoses; **TimeCE** – Time from symptom onset to coronary event (hours); **HTA** – Arterial hypertension; **DM** – Diabetes mellitus; **DMID** – Insulin-dependent diabetes; **HypLip** – Hyperlipidemia; **Smoking** – Smoking status; **Family history cardiac** – Family history of cardiovascular disease; **HBI** – History of brain ischemia; **Cancer** – History of malignancy; **Inf Lung** – Postoperative pulmonary infection (e.g. pneumonia); **Hypothyreosis** – Hypothyroidism; **HOBP** – Chronic obstructive pulmonary disease (COPD); **PVB** – Peripheral vascular disease; **Levosimendan** – Use of levosimendan for inotropic support; **StenCar** – Carotid artery stenosis. **Sedative** – Sedative drug administration (yes/no or type); **Neutro io** – Postoperative neutrophil count ($\times10^{9}$/L); **mono io** – Postoperative monocyte count ($\times10^{9}$/L); **Neu mon io** – Combined neutrophil and monocyte count ($\times10^{9}$/L); **IO INO** – Intraoperative inotropic support; **IO VP** – Intraoperative vasopressor use; **IO levosimendan** – Intraoperative levosimendan use; **IO milrinon** – Intraoperative milrinone use; **IO kortiko** – Intraoperative corticosteroid use; **IO amiodaron** – Intraoperative amiodarone use; **GIK** – Glucose-insulin-potassium infusion; **Time INO** – Duration of inotropic support (minutes); **Time VP** – Duration of vasopressor support (minutes); **Time Ins pump** – Duration of insulin pump infusion (minutes); **Sedation MV** – Sedation in combination with mechanical ventilation; **What sedative** – Type or cumulative score of sedative medication; **ARDS** – Acute respiratory distress syndrome.
